# Prevalence of dumping and hypoglycaemia symptoms after bariatric surgery: A questionnaire‐based cross‐sectional study

**DOI:** 10.1111/cob.12709

**Published:** 2024-10-11

**Authors:** Anders Jans, Eva Rask, Johan Ottosson, Eva Szabo, Erik Stenberg

**Affiliations:** ^1^ Department of Surgery, Faculty of Medicine and Health Örebro University Örebro Sweden; ^2^ University Health Care Research Centre, Faculty of Medicine and Health Örebro University Örebro Sweden

**Keywords:** bariatric surgery, dumping, hypoglycaemia, prevalence, questionnaire

## Abstract

Dumping and post‐bariatric hypoglycaemia (PBH) are side effects that occur after bariatric surgery. The aim of this study was to estimate the prevalence of dumping and PBH symptoms before Roux‐en‐Y gastric bypass (RYGB) and sleeve gastrectomy (SG) at 6 months, 1 year, 2 years and 5 years after surgery in a Swedish population. A cross‐sectional single‐centre study was performed at Lindesberg Hospital, Region Örebro County, Sweden, between 2020 and 2023. The Swedish version of the Dumping Severity Scale (DSS‐Swe) questionnaire, which includes eight items regarding dumping symptoms and six items regarding hypoglycaemia symptoms, was used. A total of 742 DSS‐Swe questionnaires were included. The average age at surgery was 42.0 years (standard deviation [SD] = 11.9), and the average body mass index was 41.8 kg/m^2^ (SD = 5.9). The surgical methods consisted of RYGB (66.3%) and SG (33.7%). The proportion of RYGB patients with highly suspected dumping increased from 4.9% before surgery to 26.3% (adjusted odds ratio [OR] = 7.35, 95% confidence interval [CI] = 3.08–17.52) at the 5‐year follow‐up. PBH symptoms increased from 1.4% before surgery to 19.3% at the 5‐year follow‐up (adjusted OR = 17.88, 95% CI = 4.07–78.54). For SG patients, no significant increase in dumping or PBH symptoms was observed. In patients with persistent type 2 diabetes (T2D), there were no cases of highly suspected hypoglycaemia following RYGB or SG. Symptoms of dumping and PBH were common after RYGB, while no clear increase was observed after SG. Persistent T2D seems to be a protective factor against PBH symptoms.

**What is already known about this subject**

- Dumping and post‐bariatric hypoglycaemia are side effects that occur after bariatric surgery and may result in a significant reduction in quality of life.

**What this study adds**

- Symptoms of dumping and hypoglycaemia are common side effects after Roux‐en‐Y gastric bypass.
- No increase of dumping and hypoglycaemia symptoms was observed after sleeve gastrectomy.
- Persistent type 2 diabetes seems to be a protective factor against hypoglycaemia symptoms.

## 1 INTRODUCTION

Globally, the proportion of people with obesity has risen gradually since 1980.^1^ Every year, more than half a million bariatric surgeries are performed worldwide.^2^ Compared to non‐surgical treatment, bariatric surgery is a more effective way to achieve long‐term weight loss in individuals with obesity and increases survival in this patient group.^3^, ^4^, ^5^ Moreover, in the case of type 2 diabetes (T2D) mellitus and concomitant obesity with a body mass index (BMI) greater than 35 kg/m^2^, bariatric surgery is more effective against diabetes than medical treatment.^6^ The most common bariatric surgical methods at present are sleeve gastrectomy (SG) and Roux‐en‐Y gastric bypass (RYGB).^2^

Dumping is a condition associated with bariatric surgery and usually occurs within the first hour after a meal because of the rapid passage of undigested food into the jejunum. Osmotic effects, the release of peptide hormones and autonomic neural responses cause gastrointestinal and vasomotor symptoms. Dumping is not related to hypoglycaemia.^7^

Post‐bariatric hypoglycaemia (PBH), historically referred to as ‘late dumping syndrome’, is a type of hyperinsulinemic hypoglycaemia with varying prevalence according to previous studies. Some studies have reported high prevalence of PBH after RYGB; however, PBH also occurs after SG and usually results in symptoms 1–3 h after a meal.^7^, ^8^, ^9^, ^10^, ^11^, ^12^, ^13^, ^14^ PBH debuts more than 1 year after bariatric surgery, and the median time between surgery and the onset of symptoms has been reported to be between 2 and 3 years.^8^, ^15^ Although PBH generally produces relatively mild symptoms, in rare cases, it can give rise to very severe conditions such as convulsions and unconsciousness.^15^ However, asymptomatic PBH appears to be common.^12^ Symptomatic dumping and hypoglycaemia after bariatric surgery result in a significant reduction in quality of life.^16^

There are several questionnaires for symptoms related to PBH, but none have been formally validated.^17^ The Dumping Severity Scale (DSS), a questionnaire that was initially constructed to measure the treatment response to octreotide in patients with dumping syndrome, was developed by Arts et al.^18^ The DSS has also been used to assess symptoms of dumping and hypoglycaemia after bariatric surgery.^19^, ^20^ The questionnaire includes eight typical symptoms of early dumping and six common symptoms of hypoglycaemia, which are graded by the patient on a Likert scale ranging from 0 to 3 points depending on the severity, or absence, of the respective symptoms. The DSS questionnaire has been translated into Swedish (DSS‐Swe), and its reliability has been tested in Swedish populations.^21^

The aim of this study was to estimate the prevalence of symptoms of dumping and hypoglycaemia before bariatric surgery, as well as at 6 months, 1 year, 2 years and 5 years after bariatric surgery, in a Swedish population using the DSS‐Swe questionnaire.

## 2 MATERIALS AND METHODS

This was a cross‐sectional study in which symptoms of dumping and hypoglycaemia were assessed at different time points before and after bariatric surgery. Patients (aged ≥18 years) who were scheduled to undergo bariatric surgery at Lindesberg Hospital or during routine follow‐up 6 months, 1 year, 2 years or 5 years after surgery during the inclusion period from September 2020 to September 2023 were invited to complete the DSS‐Swe questionnaire. To be included in the study, the ability to understand and complete the questionnaire, with or without the support of an interpreter, was needed.

Patients who underwent bariatric revision surgery and patients without scheduled surgery were excluded since they could constitute a heterogeneous subgroup that would increase the risk of introducing bias into the study. Patients who were receiving ongoing pharmacological treatment for hypoglycaemia (*n* = 2) were also excluded. Due to the COVID‐19 pandemic, many follow‐up visits were performed with telemedical assistance, limiting the number of patients available for inclusion.

Lindesberg Hospital is the centre for bariatric surgery in Region Örebro County, Sweden. The clinical strategy for the management of patients with dumping and suspected PBH at Lindesberg Hospital was unchanged during the study period.

In accordance with the findings of a previous study, highly suspected dumping was defined as having at least three symptoms with an intensity of at least two (moderate or severe), of which at least one was an autonomic symptom.^16^ Similarly, highly suspected hypoglycaemia was defined as having three symptoms with an intensity of at least two, including at least one neuroglycopenic symptom.

Patients who completed the DSS‐Swe questionnaire at more than one follow‐up time point were registered as a separate individual at each time point.

Information on baseline characteristics, surgery and follow‐up was based on data from the Scandinavian Obesity Surgery Registry (SOReg). The SOReg was launched in 2007 as a national quality and research register reporting preoperative, intraoperative and follow‐up data at 30 days and 1, 2, 5, 10 and 15 years after surgery. At present, the registry covers practically all bariatric surgical procedures in Sweden, with a very high acquisition rate and internal validity.^22^

### 2.1 Procedures

The surgical method for gastric bypass was highly standardised during the study period, with all procedures being antecolic/antegastric gastric bypass procedures with a small gastric pouch, a biliopancreatic limb of 50–70 cm and an alimentary limb of 100–120 cm.^23^ The gastro‐jejunostomy was constructed using 30 mm of a linear cutting stapler cartridge with hand‐sewn closure of the remaining defect using continuous absorbable sutures. SG was routinely performed using a 35 Fr bougie with the gastric division starting 2–5 cm from the pylorus and ending 1 cm from the angle of His. Perioperative care closely followed the Enhanced Recovery After Surgery guidelines and included early mobilisation, routine thromboprophylaxis, and the start of oral fluids on the day of surgery.^24^

### 2.2 Statistical analysis

The data are presented as the number of individuals (*n*) with percentages of patients for categorical values and as the mean ± standard deviation (SD) for continuous variables assuming a normal distribution. One‐way ANOVA was used when comparing continuous variables in more than two groups with assumed normal distributions, and the chi‐square test was used for categorical variables. Logistic regression analysis was used when comparing categorical variables with adjustments for possible confounding factors (surgical procedure, sex, BMI and type 2 diabetes) based on previous studies and the plausible impact of preoperatively available factors.^10^, ^16^

A complete case analysis was used; i.e., when there were invalidly completed questionnaire items, that section of the questionnaire (dumping section and/or hypoglycaemia section) was excluded from further analysis.

SPSS Statistics version 29 (IBM, Armonk, New York, USA) was used for all the statistical analyses.

### 2.3 Ethics

The study was approved by the Ethics Committee in Stockholm (Dnr 2020‐01257) and conducted in accordance with the ethical standards of the 1964 Helsinki Declaration and its later amendments. Informed consent was obtained from all individual participants included in the study.

## 3 RESULTS

**FIGURE 1:**
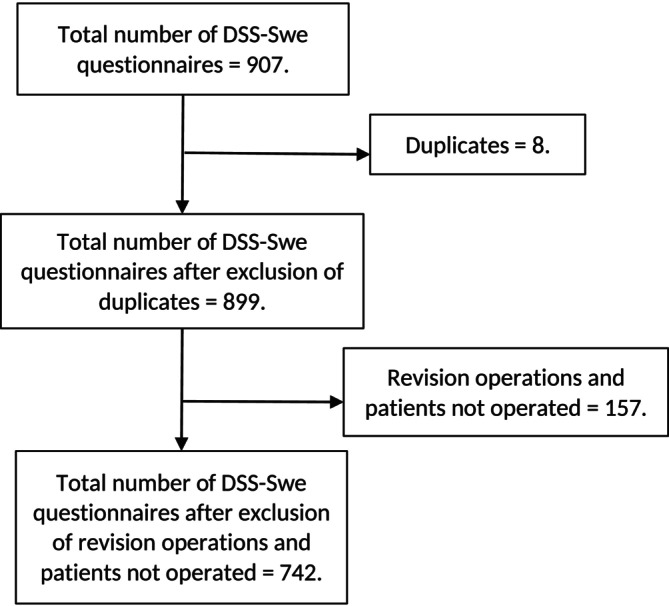
Study flowchart. DSS‐Swe, the Swedish version of the Dumping Severity Scale.

A total of 907 DSS‐Swe questionnaires were completed. After removing duplicates and questionnaires from excluded patients, 742 questionnaires from 634 unique individuals remained. Ninety‐nine patients completed the DSS‐Swe questionnaire at two or more separate time points (Figure 1).

The average age at surgery was 42.0 years (SD = 11.9), and the surgical methods were RYGB (66.3%) and SG (33.7%) (Table 1). The baseline characteristics of each subgroup stratified according to the time point in relation to surgery are presented in Table S1. The proportion of patients with symptoms consistent with highly suspected dumping increased from 5.3% before surgery to 20.3% at the 5‐year follow‐up, with an odds ratio (OR) of 5.08 (95% confidence interval [CI] = 2.41–10.69) after adjustment for surgical procedure, sex, BMI and T2D at baseline (Table 2). The proportion of patients with highly suspected hypoglycaemia increased from 1.6% to 14.8%, with an adjusted OR of 12.19 (95% CI = 3.57–41.61) (Table 3).

**TABLE 1:** Baseline characteristics.

Characteristic	Missing data	
Individuals	0 (0.0%)	742 (100.0%)
Preoperatively, *n* (%)		190 (25.6%)
6 months post‐operatively, *n* (%)		60 (8.1%)
1 year post‐operatively, *n* (%)		169 (22.8%)
2 years post‐operatively, *n* (%)		151 (20.4%)
5 years post‐operatively, *n* (%)		172 (23.2%)
Preoperative BMI, mean ± SD, kg/m^2^	0 (0.0%)	41.8 ± 5.9
Age at surgery, mean ± SD, years	0 (0.0%)	42.0 ± 11.9
Procedure, *n* (%)	0 (0.0%)	
Gastric bypass, *n* (%)		492 (66.3%)
Sleeve gastrectomy, *n* (%)		250 (33.7%)
Sex	0 (0.0%)	
Female, *n* (%)		576 (77.6%)
Male, *n* (%)		166 (22.4%)
Comorbidity prior to surgery		
Sleep apnoea, *n* (%)	0 (0.0%)	136 (18.3%)
Hypertension, *n* (%)	0 (0.0%)	191 (25.7%)
Dyslipidaemia, *n* (%)	0 (0.0%)	53 (7.1%)
Dyspepsia/gastroesophageal reflux disease, *n* (%)	0 (0.0%)	60 (8.1%)
Depression, *n* (%)	0 (0.0%)	104 (14.0%)
Previous pulmonary embolus/deep venous thrombosis, *n* (%)	0 (0.0%)	12 (1.6%)
Type 2 diabetes mellitus prior to surgery, *n* (%)	0 (0.0%)	97 (13.1%)
Glycosylated haemoglobin A1c preoperatively, mmol/mol, mean ± SD	5 (0.7%)	40.0 ± 8.6
Education	17 (2.3%)	
Primary education ≤9 years, *n* (%)		47 (6.5%)
Secondary education 10–12 years, *n* (%)		520 (71.7%)
Higher education, *n* (%)		158 (21.8%)

Abbreviations: BMI, body mass index; SD, standard deviation.

**TABLE 2:** Prevalence of dumping symptoms in relation to follow‐up time point.

	High suspicion of dumping, *n* (%)				
Follow‐up time point	Missing data		OR (95% CI)^a^	Adj OR (95% CI)^b^	*p* ^b^
All patients (*n* = 742)	0 (0.0%)	102 (13.7%)			
Preoperatively (*n* = 190)	0 (0.0%)	10 (5.3%)	Reference	Reference	Reference
6 months post‐operatively (*n* = 60)	0 (0.0%)	8 (13.3%)	2.77 (1.04–7.38)	3.11 (1.15–8.41)	0.026
1 year post‐operatively (*n* = 169)	0 (0.0%)	26 (15.4%)	3.27 (1.53–7.01)	3.71 (1.72–8.01)	<0.001
2 years post‐operatively (*n* = 151)	0 (0.0%)	23 (15.2%)	3.23 (1.49–7.03)	3.67 (1.68–8.06)	0.001
5 years post‐operatively (*n* = 172)	0 (0.0%)	35 (20.3%)	4.60 (2.20–9.61)	5.08 (2.41–10.69)	<0.001
Gastric bypass (*n* = 492)	0 (0.0%)	79 (16.1%)			
Preoperatively (*n* = 142)	0 (0.0%)	7 (4.9%)	Reference	Reference	Reference
6 months post‐operatively (*n* = 36)	0 (0.0%)	4 (11.1%)	2.41 (0.67–8.74)	2.46 (0.67–9.03)	0.17
1 year post‐operatively (*n* = 103)	0 (0.0%)	17 (16.5%)	3.81 (1.52–9.57)	3.84 (1.52–9.69)	0.004
2 years post‐operatively (*n* = 93)	0 (0.0%)	20 (21.5%)	5.28 (2.13–13.08)	5.46 (2.19–13.58)	<0.001
5 years post‐operatively (*n* = 118)	0 (0.0%)	31 (26.3%)	6.87 (2.90–16.29)	7.35 (3.08–17.52)	<0.001
Sleeve gastrectomy (*n* = 250)	0 (0.0%)	23 (9.2%)			
Preoperatively (*n* = 48)	0 (0.0%)	3 (6.3%)	Reference	Reference	Reference
6 months post‐operatively (*n* = 24)	0 (0.0%)	4 (16.7%)	3.00 (0.61–14.67)	3.11 (0.62–15.71)	0.17
1 year post‐operatively (*n* = 66)	0 (0.0%)	9 (13.6%)	2.37 (0.61–9.26)	2.67 (0.67–10.66)	0.17
2 years post‐operatively (*n* = 58)	0 (0.0%)	3 (5.2%)	0.82 (0.16–4.25)	0.88 (0.17–4.65)	0.88
5 years post‐operatively (*n* = 54)	0 (0.0%)	4 (7.4%)	0.82 (0.26–5.66)	1.23 (0.26–5.83)	0.80

Abbreviations: BMI, body mass index; CI, confidence interval; OR, odds ratio.

^a^ Non‐adjusted logistic regression.

^b^ Logistic regression adjusted for surgical procedure, sex, BMI and diabetes type 2 at baseline.

**TABLE 3:** Prevalence of hypoglycaemia symptoms in relation to follow‐up time point.

	High suspicion of hypoglycaemia, *n* (%)				
Follow‐up time point	Missing data		OR (95% CI)^a^	Adj OR (95% CI)^b^	*p* ^b^
All patients (*n* = 742)	14 (1.9%)	53 (7.3%)			
Preoperatively (*n* = 190)	2 (1.1%)	3 (1.6%)	Reference	Reference	Reference
6 months post‐operatively (*n* = 60)	1 (1.7%)	2 (3.4%)	2.16 (0.35–13.27)	2.58 (0.42–15.98)	0.31
1 year post‐operatively (*n* = 169)	1 (0.6%)	8 (4.8%)	3.08 (0.80–11.82)	3.53 (0.92–13.62)	0.067
2 years post‐operatively (*n* = 151)	0 (0.0%)	16 (10.6%)	7.31 (2.09–25.58)	8.60 (2.44–30.39)	<0.01
5 years post‐operatively (*n* = 172)	10 (5.8%)	24 (14.8%)	10.73 (3.17–36.34)	12.19 (3.57–41.61)	<0.01
Gastric bypass (*n* = 492)	13 (2.6%)	41 (8.6%)			
Preoperatively (*n* = 142)	2 (1.4%)	2 (1.4%)	Reference	Reference	Reference
6 months post‐operatively (*n* = 36)	1 (2.8%)	1 (2.9%)	2.03 (0.18–23.04)	2.13 (0.19–24.39)	0.54
1 year post‐operatively (*n* = 103)	1 (1.0%)	4 (3.9%)	2.82 (0.51–15.68)	2.83 (0.51–15.83)	0.24
2 years post‐operatively (*n* = 93)	0 (0.0%)	13 (14.0%)	11.21 (2.47–50.96)	11.74 (2.57–53.63)	0.001
5 years post‐operatively (*n* = 118)	0 (0.0%)	21 (19.3%)	16.47 (3.77–71.96)	17.88 (4.07–78.54)	<0.001
Sleeve gastrectomy (*n* = 250)	1 (0.4%)	12 (4.8%)			
Preoperatively (*n* = 48)	0 (0.0%)	1 (2.1%)	Reference	Reference	Reference
6 months post‐operatively (*n* = 24)	0 (0.0%)	1 (4.2%)	2.04 (0.12–34.16)	2.66 (0.16–45.70)	0.50
1 year post‐operatively (*n* = 66)	0 (0.0%)	4 (6.1%)	3.03 (0.33–28.03)	4.16 (0.44–39.10)	0.21
2 years post‐operatively (*n* = 58)	0 (0.0%)	3 (5.2%)	2.56 (0.26–25.49)	3.33 (0.33–33.69)	0.31
5 years post‐operatively (*n* = 54)	1 (1.9%)	3 (5.7%)	2.82 (0.28–28.07)	2.98 (0.30–30.00)	0.35

Abbreviations: BMI, body mass index; CI, confidence interval; OR, odds ratio.

^a^ Non‐adjusted logistic regression.

^b^ Logistic regression adjusted for surgical procedure, sex, BMI and diabetes type 2 at baseline.

The proportion of highly suspected dumping in RYGB patients increased from 4.9% before surgery to 26.3% (adjusted OR = 7.35, 95% CI = 3.08–17.52) at the 5‐year follow‐up. For SG patients, no significant increase in dumping symptoms was observed during follow‐up compared to baseline.

The proportion of RYGB patients with highly suspected PBH increased from 1.4% before surgery to 19.3% (adjusted OR = 17.88, 95% CI = 4.07–78.54) at the 5‐year follow‐up. In patients who underwent SG, there was a nonsignificant increase in the prevalence of PBH symptoms from 2.1% to 5.7% (adjusted OR = 2.96, 95% CI = 0.30–33.00). For patients who underwent RYGB, the 2‐year follow‐up was the first time a significant increase in PBH symptoms was observed (Table 3). After SG, no such increase was observed.

At the 5‐year follow‐up, there was a significantly greater risk of developing dumping symptoms (OR = 4.90, 95% CI = 1.60–15.02 when adjusted for BMI, sex and T2D at baseline) and PBH symptoms (adjusted OR = 5.80, 95% CI = 1.58–21.34) in RYGB patients than in SG patients. None of the patients with persistent T2D reported symptoms consistent with highly suspected PBH after RYGB or after SG (Table 4).

**TABLE 4:** Proportion of patients with high suspicion of hypoglycaemia symptoms at different follow‐up time points.

Patient characteristic	Baseline	1 year	2 years	5 years	OR (95% CI)^a^	Adj OR (95% CI)^b^	*p* ^b^
All surgical methods							
All patients	1.6% (3/188)	4.8% (8/168)	10.6% (16/151)	14.8% (24/162)	10.73 (3.17–36.34)	12.19 (3.57–41.61)	<0.001
Patients with T2D at baseline	0.0% (0/14)	3.6% (1/28)	9.1% (2/22)	11.1% (2/18)	–	–	–
Patients without T2D at baseline	1.7% (3/174)	5.0% (7/140)	10.9% (14/129)	15.3% (22/144)	10.28 (3.01–35.11)	11.09 (3.22–38.12)	<0.001
Patients with T2D at follow‐up	0.0% (0/14)	0.0% (0/16)	0.0% (0/13)	0.0% (0/10)	–	–	–
Patients without T2D at follow‐up	1.7% (3/174)	5.6% (8/144)	12.3% (16/130)	14.7% (20/136)	9.83 (8.86–33.83)	10.35 (2.97–35.95)	<0.001
Patients with T2D at baseline, but remission at follow‐up	–	9.1% (1/11)	22.2% (2/9)	12.5% (1/8)	–	–	–
Gastric bypass							
All patients	1.4% (2/140)	3.9% (4/102)	14.0% (13/93)	19.3% (21/109)	16.47 (3.77–71.97)	17.88 (4.07–78.54)	<0.001
Patients with T2D at baseline	0.0% (0/10)	0.0% (0/15)	18.2% (2/11)	15.4% (2/13)	–	–	–
Patients without T2D at baseline	1.5% (2/130)	4.6% (4/87)	13.4% (11/82)	19.8% (19/96)	15.79 (3.58–69.97)	16.20 (3.66–71.76)	<0.001
Patients with T2D at follow‐up	0.0% (0/10)	0.0% (0/9)	0.0% (0/7)	0.0% (0/6)	–	–	–
Patients without T2D at follow‐up	1.5% (2/130)	4.5% (4/89)	16.0% (13/81)	18.5% (17/92)	14.51 (3.26–64.54)	14.74 (3.28–66.26)	<0.001
Patients with T2D at baseline, but remission at follow‐up	–	0.0% (0/6)	50.0% (2/4)	16.7% (1/6)	–	–	–
Sleeve gastrectomy							
All patients	2.1% (1/48)	6.1% (4/66)	5.2% (3/58)	5.7% (3/53)	2.82 (0.28–28.07)	2.98 (0.30–30.00)	0.35
Patients with T2D at baseline	0.0% (0/4)	7.7% (1/13)	0.0% (0/11)	0.0% (0/5)	–	–	–
Patients without T2D at baseline	2.3% (1/44)	5.7% (3/53)	6.4% (3/47)	6.3% (3/48)	2.86 (0.29–28.63)	2.97 (0.29–29.95)	0.36
Patients with T2D at follow‐up	0.0% (0/4)	0.0% (0/7)	0.0% (0/6)	0.0% (0/4)	–	–	–
Patients without T2D at follow‐up	2.3% (1/44)	7.3% (4/55)	6.1% (3/49)	6.8% (3/44)	3.15 (0.31–31.48)	3.04 (0.30–31.04)	0.35
Patients with T2D at baseline, but remission at follow‐up	–	20.0% (1/5)	0.0% (0/5)	0.0% (0/2)	–	–	–

Abbreviations: BMI, body mass index; CI, confidence interval; OR, odds ratio; T2D, diabetes mellitus type 2.

^a^ Non‐adjusted logistic regression. OR for high suspicion of hypoglycaemia at 5 years compared to baseline.

^b^ Logistic regression adjusted for surgical procedure, sex, BMI and diabetes type 2 at baseline. OR for high suspicion of hypoglycaemia at 5 years compared to baseline.

## 4 DISCUSSION

A clear increase in the prevalence of symptoms consistent with dumping and PBH was reported by patients who underwent RYGB, while no major difference was reported among patients who underwent SG. This finding is consistent with the results of previous studies.^10^, ^19^

There was also a clear difference between patients with and without T2D at follow‐up. None of the patients with T2D at follow‐up reported pronounced symptoms of hypoglycaemia. Thus, T2D appears to be a protective factor against the development of hypoglycaemic symptoms, which may be explained by the decreased insulin sensitivity in patients with persistent T2D. Insulin sensitivity has indeed been reported to be greater in patients who suffer from PBH.^12^

A significant increase in hypoglycaemic symptoms after RYGB was first demonstrated at the 2‐year follow‐up. These findings support data from previous studies suggesting that the median time between surgery and the development of symptomatic PBH is usually 2–3 years.^8^, ^15^

In the literature, there are several differences in the categorisation of dumping symptoms between abdominal symptoms and autonomic symptoms. Likewise, the categorisation of autonomic symptoms and neuroglycopenic symptoms associated with hypoglycaemia differs to some extent. In this study, the categorisation described in the ‘International Consensus on the Diagnosis and Management of Dumping Syndrome’ by Scarpellini et al. was used.^7^ Depending on how the cut‐off criteria for highly suspected dumping or PBH symptoms are defined, the percentage of patients who meet the criteria varies. When comparing different surgical methods with the same cut‐off criteria for symptoms, this problem is reduced. However, the proportions of RYGB‐operated patients with highly suspected dumping and PBH symptoms in this study were consistent with those of another previously conducted study based on the DSS questionnaire.^16^

The lack of a clear definition of symptomatic PBH prevents validation. Consequently, this also means that the DSS‐Swe questionnaire, on which this study was based, has not been formally validated. However, there are no other existing questionnaires for post‐bariatric hypoglycaemic symptoms that have been formally selected either.^17^ It is also known that the agreement between measured hypoglycaemia and perceived symptoms is very poor.^12^

Hypoglycaemic symptoms are likely to be underestimated after RYGB. One reason for this is that PBH symptoms are often misinterpreted as dumping symptoms.^25^ An increased focus on such symptoms may result in earlier detection and better treatment for this group of patients. Therefore, the use of questionnaires such as the DSS or DSS‐Swe could be valuable. With these questionnaires, symptoms that give rise to the suspicion of PBH are likely to be captured more systematically than during a regular follow‐up visit. Nutritional therapy remains the key cornerstone for the successful prevention of hypoglycaemia in patients with PBH. In fact, the vast majority of patients can be successfully managed with dietary modifications.^11^ Limiting high‐glycaemic‐index carbohydrates reduces the postprandial glucose spike and insulin response preceding hypoglycaemia. Furthermore, adequate protein and fat intake will contribute to the balance needed to achieve protein and calorie goals.^26^ However, some patients have PBH that is refractory to dietary modifications. In those cases, pharmacotherapy with drugs such as α‐glucosidase inhibitors, GLP‐1 receptor agonists and somatostatin analogues may be useful.^11^, ^27^ Several of the patients in this study who reported pronounced symptoms in the DSS‐Swe questionnaire were offered diagnostic measures and were treated if appropriate. It is important to realise that the DSS‐Swe should not be used for diagnostic purposes, but rather as a screening tool with the aim of identifying those individuals who should be investigated further.

The primary aim of the present study was to provide a descriptive analysis of the risk of developing symptoms consistent with dumping and hypoglycaemia after bariatric surgery in a Swedish population using the DSS‐Swe questionnaire. The Swedish bariatric surgical population is similar to those of many other European countries. With caution, the results of this study could therefore be generalised to patients undergoing bariatric surgery in other parts of Europe as well.

## 5 LIMITATIONS

Several limitations need to be acknowledged. At the start of the study, all patients considered for bariatric surgery or planned for postoperative follow‐up were offered the opportunity to participate, but the registration of those who declined participation was not recorded. Therefore, it was not possible to perform a sensitivity analysis of patients who declined participation. However, the characteristics of the patients in the present study are similar to those of the bariatric surgical population in Sweden.^28^

Information on diabetes at the various follow‐up time points was obtained from the SOReg. Despite reaching adequate glucose control, patients are sometimes encouraged by healthcare providers to continue certain diabetes drugs, such as metformin.^29^ Since the definition of diabetes in the SOReg includes pharmacological treatment, T2D remission may therefore be underestimated in some cases.

Due to the nature of cross‐sectional studies, it was decided not to exclude patients who reported symptoms of dumping or hypoglycaemia in the preoperative setting, as retrospective questions in the questionnaire would introduce a risk for recall bias. Nevertheless, defining the preoperative level as ‘baseline’ allowed the estimation of symptom changes at a group level. The results of the present study need to be verified in a prospective cohort.

### 5.1 Conclusion

Symptoms of dumping and PBH were more common after RYGB, while no clear increase was observed after SG. Persistent T2D seems to be a protective factor against PBH symptoms.

## AUTHOR CONTRIBUTIONS

A.J., E.R., J.O., E.Sz. and E.St. designed the study. A.J. and E.St. conducted data analysis and data interpretation. All authors were involved in writing the manuscript, and had final approval of the submitted version.

## CONFLICT OF INTEREST STATEMENT

E.St. has received lecturing fees from MSD and consulting fees from the Swedish National Board of Health and Welfare and Johnson & Johnson Medical (paid to the institution). The remaining authors declare no conflict of interest.

## Supporting information


**Table S1.** Baseline characteristics.
