# Supplementary material for: Prevalence of dumping and hypoglycaemia symptoms after bariatric surgery: A questionnaire‐based cross‐sectional study
Source: Clin Obes. 2024 Oct 11;15(1):e12709. doi: 10.1111/cob.12709 (PMC11706738; doi:10.1111/cob.12709)
Supplement: Supplementary file 1 — Table S1. Baseline characteristics. [file COB-15-e12709-s001.pdf]

Prevalence of dumping and hypoglycemia symptoms after bariatric surgery: A questionnaire-based cross-sectional study.

Anders Jans, M.D.[a], Eva Rask, M.D., Ph.D.[b], Johan Ottosson, M.D., Ph.D.[a], Eva Szabo, M.D., Ph.D.[a], Erik Stenberg, M.D., Ph.D.[a]

Affiliation: [a] Department of Surgery, Faculty of Medicine and Health, Örebro University, Örebro, Sweden.

[b] University Health Care Research Centre, Faculty of Medicine and Health, Örebro University, Örebro, Sweden.

Correspondence: Anders Jans, Department of Surgery, Örebro University Hospital, SE-70185 Örebro, Sweden. E-mail: anders.jans@regionorebrolan.se, telephone: +46(0)196021666, fax +46(0)19125439

Supplementary Table S1. Baseline characteristics.	All patients (n=742)		Pre-operatively (n=190)		6 months post-op (n=60)		1 year post-op (n=169)		2 years post-op (n=151)		5 years post-op (n=172)		P value[a]
**Characteristic**	**Missing data**		**Missing data**		**Missing data**		**Missing data**		**Missing data**		**Missing data**		
Preoperative BMI, mean ± SD, kg/m^2	0 (0.0%)	41.8 ± 5.9	0 (0.0%)	41.9 ± 5.5	0 (0.0%)	41.7 ± 6.4	0 (0.0%)	42.1 ± 6.7	0 (0.0%)	41.6 ± 6.0	0 (0.0%)	41.6 ± 5.4	0.91
Age at surgery, mean ± SD, years	0 (0.0%)	42.0 ± 11.9	0 (0.0%)	40.3 ± 10.7	0 (0.0%)	42.7 ± 11.1	0 (0.0%)	43.1 ± 12.3	0 (0.0%)	43.0 ± 11.8	0 (0.0%)	41.6 ± 13.1	0.15
Procedure, n (%)	0 (0.0%)		0 (0.0%)		0 (0.0%)		0 (0.0%)		0 (0.0%)		0 (0.0%)		0.024
Gastric bypass, n (%)		492 (66.3%)		142 (74.7)		36 (60.0%)		103 (60.9%)		93 (61.6%)		118 (68.6%)	
Sleeve gastrectomy, n (%)		250 (33.7%)		48 (25.3)		24 (40.0%)		66 (39.1%)		58 (38.4%)		54 (31.4%)	
Sex	0 (0.0%)		0 (0.0%)		0 (0.0%)		0 (0.0%)		0 (0.0%)		0 (0.0%)		0.30
Female, n (%)		576 (77.6%)		158 (83.2%)		46 (76.7%)		130 (76.9%)		112 (74.2%)		130 (75.6%)	
Male, n (%)		166 (22.4%)		32 (16.8%)		14 (23.3%)		39 (23.1%)		39 (25.4%)		42 (24.4%)	
Comorbidity prior to surgery													
Sleep apnea, n (%)	0 (0.0%)	136 (18.3%)	0 (0.0%)	25 (13.2%)	0 (0.0%)	16 (26.7%)	0 (0.0%)	43 (25.4%)	0 (0.0%)	31 (20.5%)	0 (0.0%)	21 (12.2%)	0.002
Hypertension, n (%)	0 (0.0%)	191 (25.7%)	0 (0.0%)	41 (21.6%)	0 (0.0%)	19 (31.7%)	0 (0.0%)	47 (27.8%)	0 (0.0%)	43 (28.5%)	0 (0.0%)	41 (23.8%)	0.39
Dyslipidemia, n (%)	0 (0.0%)	53 (7.1%)	0 (0.0%)	9 (4.7%)	0 (0.0%)	7 (11.7%)	0 (0.0%)	13 (7.7%)	0 (0.0%)	13 (8.6%)	0 (0.0%)	11 (6.4%)	0.38
Dyspepsia / Gastroesophageal reflux disease, n (%)	0 (0.0%)	60 (8.1%)	0 (0.0%)	15 (7.9%)	0 (0.0%)	7 (11.7%)	0 (0.0%)	18 (10.7%)	0 (0.0%)	12 (7.9%)	0 (0.0%)	8 (4.7%)	0.26
Depression, n (%)	0 (0.0%)	104 (14.0%)	0 (0.0%)	34 (17.9%)	0 (0.0%)	7 (11.7%)	0 (0.0%)	32 (18.9%)	0 (0.0%)	16 (10.6%)	0 (0.0%)	15 (8.7%)	0.021
Previous pulmonary embolus / Deep venous thrombosis, n (%)	0 (0.0%)	12 (1.6%)	0 (0.0%)	4 (2.1%)	0 (0.0%)	2 (3.3%)	0 (0.0%)	2 (1.2%)	0 (0.0%)	2 (1.3%)	0 (0.0%)	2 (1.2%)	0.75
Type 2 diabetes mellitus prior to surgery, n (%)	0 (0.0%)	97 (13.1%)	0 (0.0%)	14 (7.4%)	0 (0.0%)	15 (25.0%)	0 (0.0%)	28 (16.6%)	0 (0.0%)	22 (14.6%)	0 (0.0%)	18 (10.5%)	0.003
Glycosylated Hemoglobin A1c pre-operatively, mmol/mol, mean ± SD	5 (0.7%)	40.0 ± 8.6	3 (1.6%)	38.8 ± 5.3	0 (0.0%)	41.9 ± 10.4	0 (0.0%)	41.0 ± 9.2	1 (0.7%)	40.6 ± 10.1	1 (0.6%)	39.0 ± 8.6	0.015
Education	17 (2.3%)		2 (1.1%)		0 (0.0%)		3 (1.8%)		3 (2.0%)		9 (5.2%)		0.25
Primary education ≤9 yrs, n (%)		47 (6.5%)		13 (6.9%)		6 (10.0%)		15 (9.0%)		7 (4.7%)		6 (3.7%)	
Secondary education 10-12 yrs, n (%)		520 (71.7%)		133 (70.7%)		46 (86.7%)		120 (72.3%)		107 (72.3%)		114 (69.9%)	
Higher education, n (%)		158 (21.8%)		42 (22.3%)		8 (13.3%)		31 (18.7%)		34 (23.0%)		43 (26.4%)	

BMI = body mass index; SD = standard deviation.

[a] P value for comparison between all different follow-up groups. One-way ANOVA was used for continuous variables with assumed normal distribution. Chi-Square was used for categorical variables.
